# Cholesterol-Induced M4-Like Macrophages Recruit Neutrophils and Induce NETosis

**DOI:** 10.3389/fimmu.2021.671073

**Published:** 2021-05-03

**Authors:** Ana C. Maretti-Mira, Lucy Golden-Mason, Matthew P. Salomon, Mariana J. Kaplan, Hugo R. Rosen

**Affiliations:** ^1^Department of Medicine, Keck School of Medicine, Gastroenterology & Hepatology, Research Center for Liver Disease, University of Southern California (USC), Los Angeles, CA, United States; ^2^Systemic Autoimmunity Branch, National Institute of Arthritis and Musculoskeletal and Skin Diseases (NIAMS), National Institutes of Health (NIH), Bethesda, MD, United States

**Keywords:** macrophages, Kupffer cells, liver, low-density lipoprotein (LDL), non-alcoholic steatohepatitis (NASH), neutrophil, NETosis, transcriptomic (RNA-Seq)

## Abstract

The liver is the central organ for cholesterol synthesis and homeostasis. The effects of dietary cholesterol on hepatic injury, mainly of oxidized low-density lipoproteins (OxLDL), are not fully understood. Here, we show that the degree of cholesterol oxidation had different impacts on the global gene expression of human M2-like macrophages, with highly oxidized LDL causing the most dramatic changes. M2-like macrophages and Kupffer cells undergo M4-like polarization, decreasing the expression of important markers, such as IL10, MRC1, and CD163. These cells also displayed functional changes, with reduced phagocytic capacity, increased neutrophil recruitment, and more effective neutrophil extracellular traps (NETs) induction. Our findings provide a link between LDL oxidation and modification of peripheral and liver macrophage function.

## Introduction

The liver is the central organ for cholesterol synthesis and homeostasis and emerging evidence points towards the central role of cholesterol and especially oxidized low-density lipoproteins (OxLDL) in hepatic inflammation, contributing to the development of non-alcoholic steatohepatitis (NASH), fibrosis and cirrhosis (1, 2). Although there is a clear association between obesity and NASH, dietary cholesterol can also trigger innate immune-mediated injury even in non-obese humans and rodents (1, 3).

The liver is home to the largest population of tissue-resident macrophages in the body, i.e., Kupffer cells (KCs), constituting 20-25% of the non-parenchymal cells. Under conditions of stress, infiltrating macrophages contribute to the hepatic macrophage (4). Hepatic macrophages are a dynamic cell population, able to polarize their phenotype according to microenvironment factors. The M1 phenotype provides a primary source of proinflammatory cytokines and chemokines, while the M2/alternative phenotype participates in inflammation resolution and wound healing (5). In addition to the classically and alternatively activated macrophages, a new phenotype known as M4 has been described in atherosclerotic lesions. These so-called M4 macrophages are activated by CXCL4 or CXCL4L1 from resting (M0/M2-like) macrophages, showing downregulation of CD163, MRC1, HMOX1 (not observed in CXCL4L1-induced cells), and HLA, while maintaining or increasing CXCL8 and CCL2 expression levels (6). Once polarized, the M4 macrophages present a decreased phagocytotic capacity, and an increased cholesterol efflux (7).

OxLDL is the extracellular source of reactive oxygen species and initiates a dose-dependent increase in intracellular oxidative stress (8, 9). OxLDL, an endogenous ligand that functions as a TLR agonist (10), is taken up by macrophages and is known to trigger the production of cytokines (11). Prospective data in humans indicate that elevated levels of serum oxLDL increase the risk of future NAFLD development, at same time that higher LDL oxidation scores were directly correlated with fatty liver (12). Murine models of chronic high-fat diet demonstrate that intravenous (13) administration of oxLDL results in increased accumulation of inflammatory cells and severe hepatocellular injury including progression to advanced fibrosis (8). The precise relationship between oxLDL, macrophages, and other innate immune cells in NASH remains incompletely characterized. Here, we demonstrate that monocyte-derived macrophages display transcriptional responses that vary according to the oxidized state of LDL, including differential expression of processes involved in cholesterol metabolism, oxidative stress response, and innate inflammation; both peripheral and liver-resident macrophages exposed to highly oxidized LDL shift towards an inflammatory M4 phenotype that leads to recruitment of neutrophils and formation of neutrophil extracellular traps (NETs), which are extracellular scaffolds comprised of nuclear DNA studded with histones and granule proteins representing in many cases a process of cell death, i.e., NETosis, distinct from apoptosis and necrosis (14–18). Collectively, these findings provide novel insights into understanding the impact of LDL oxidation on hepatic innate immunity.

## Materials and Methods

### Experimental Procedures

This study and protocols were approved by the University of Southern California Institutional Review Board and abide by the Declaration of Helsinki principles.

### Cell Sources

PBMCs were collected from five healthy donors. Blood was collected in Vacutainer CPT Citrate tubes (BD Biosciences) and processed according to manufacturer’s instructions. Cells were stored in liquid nitrogen until use.

Normal human Kupffer cells (KCs) were commercially obtained from LifeNet Health. Selection criteria included BMI ranging from 19 to 27, fibrosis grade =0, and NAS score < 1.

Normal human neutrophils were obtained from one healthy donor, and cells were isolated using MACSxpress^®^ Whole Blood Neutrophil Isolation Kit, human (Miltenyi) and used fresh for chemotaxis and NETosis assays.

### Macrophage Differentiation and Stimulation

CD14^+^ monocytes were isolated from five healthy control PBMCs using the CD14 MicroBeads human kit according to the manufacturer’s protocol (Miltenyi). CD14^+^ monocytes were plated at 10^6^ cells/well in 6-well plate in RPMI 1640 with 50ng/mL of M-CSF plus 5% FBS for 5 days at 37°C/5% CO_2_. After differentiation, M2-like macrophages were stimulated for 24h with RPMI + 5%FBS media plus PBS (unstimulated control), or 50μg/mL of native low-density lipoprotein (Kalen Biomedical LLC), medium oxidized LDL (Kalen Biomedical LLC), or high oxidized LDL (Kalen Biomedical LLC). Cells were then harvested in Buffer RLT (Qiagen) + 1% 2-Mercaptoethanol (Sigma-Aldrich) and stored at -80°C until processing.

### Bulk Low-Pass RNAseq and Data Analysis

M2-like macrophages RNA was isolated using RNeasy Mini kit (Qiagen) and RNA integrity was analyzed by 2100 Expert Bioanalyzer System (Agilent), using the Agilent RNA 6000 Pico Kit (Agilent). Libraries were simultaneously prepared from extracted total RNA using Illumina Truseq Stranded mRNA library preparation kit according to the manufacturer’s protocol (Illumina). Prepared libraries were sequenced on the Illumina Nextseq500 at 3 million reads per sample at 2x75 cycles. Libraries were prepared and sequenced by the USC Molecular Genomics Core.

The raw sequencing reads were first checked for overall quality and adapter contamination using FastQC (https://www.bioinformatics.babraham.ac.uk/projects/fastqc/) prior to downstream analysis. Reads were then used to qualify transcript abundances with *Salmon* (19) using the GENCODE version 31 reference including only protein coding genes. The resulting transcript abundances were summarized to gene level counts using functions in the Bioconductor *package tximport* (20). Significantly differentially expressed genes were identified using the Bioconductor package *DESeq2* (21) with a significance threshold of FDR < 0.1 and fold change of at least 1.5 when compared to paired control (PBS) samples.

We used the Ingenuity Pathway Analysis (IPA) (Qiagen_https://www.qiagenbioinformatics.com/products/ingenuitypathway-analysis) to determine pathways, and the IPA Biomarker Tool to select the biomarker candidates (22). GeneMANIA (https://genemania.org/) was used to evaluate the strength of the networks, with following criteria: (i) automatically selection of weighting method, (ii) no additional genes or attributes, and considering (iii) co-expression, (iv) co-localization, (v) genetic and (vi) genetic and physical interactions, (vii) common pathways, (viii) shared protein domain and (ix)predicted interaction (23). The platform Gene Ontology (http://geneontology.org) was used to identify biological processes (24), using significance threshold of FDR < 0.1. The software Cluster 3.0 (http://bonsai.hgc.jp/~mdehoon/software/cluster/) was used to hierarchically cluster genes and samples (25), and Java TreeView v11.6r4 (http://jtreeview.sourceforge.net) was used to visualize the clusters and generate cluster figures (26). The RNAseq files generated for this manuscript are all deposited in the Gene Expression Omnibus (GEO) with the access number GSE160200.

### Kupffer Cell Stimulation

Kupffer cells from 5 healthy controls were plated at 10^5^ cells/well in 24 well plates, in RPMI 1640 (phenol-red free) plus 5% FBS, and additional stimuli was added as follows: PBS, 50µg/mL of native LDL or high oxLDL. Cells were incubated for 24h. Supernatant (conditioned media) was saved at -20°C and cells were harvested in buffer RLT + 1% 2-mercaptoethanol. Conditioned media was used for the chemotaxis assay and RNA was used for gene expression analysis.

For the NETosis assay, Kupffer cells from 3 healthy donors were plated at 10^5^ cells/well in 24 well plates, in RPMI 1640 (phenol-red free) plus 0.5% human serum albumin, and additional stimuli was added: PBS, 50µg/mL of native LDL or high oxLDL. Cells were incubated for 18h. Conditioned media were stored at -20°C until use.

### qPCR

Kupffer cell RNA was isolated using RNeasy Mini kit (Qiagen) and cDNA conversion was done using QuantiTect Reverse Transcription Kit (Qiagen). The QuantiTect Primer Assays (Qiagen) selected for this study targeted CXCL8 (Cat# QT00000322), IL10 (Cat# QT00041685), CD163 (Cat# QT00074641), and MRC1 (Cat# QT00012810). For housekeeping gene, we used RRN18s (Cat#QT00199367). Reactions were performed using RT² SYBR Green ROX qPCR Mastermix (Qiagen) for 40 cycles on StepOne™ Real-Time PCR System (Thermo Fisher Scientific). Gene expression from target genes were obtained by delta-delta-Ct method.

### Chemotaxis Assay

For the chemotaxis assay, we used the IncuCyte S3 System. For this assay, we used the supernatant of KCs stimulated for 24h with native LDL or high oxLDL. Incucyte^®^ Clearview 96-Well Chemotaxis Plates (Essen Biosciences) were coated with Matrigel^®^ Matrix (Corning). In the bottom chamber, we added 200µL of the following attractants: (a) RPMI 1640 (phenol-red free) + 5% FBS, (b) 1 µM fMLP, (c) 25µg/mL native LDL, (d) 25µg/mL high oxLDL, (e) conditioned media KCs + PBS, (f) conditioned media KCs + native LDL, and (g) conditioned media KCs + high oxLDL. On top chamber, we added 5x10^3^ neutrophils resuspended in RPMI 1640 (phenol-red free) plus 0.5% human serum albumin. Plates were placed into IncuCyte S3 and images were acquired every 1 hour, for 24 hours. Each condition had 4 replicates.

### NETosis Assay

For the NETosis assay, we used the IncuCyte S3 System. Normal human neutrophils were resuspended to 10^5^ cells/mL in RPMI 1640 (phenol red-free, serum-free) medium with 250nM Incucyte^®^ Cytotox Green Dye (Essen Biosciences). We added 50µL of cells suspension (2x10^4^) per wells into Incucyte^®^ Imagelock 96-well plates (Essen Biosciences). Then, we added 50µL of: (a) RPMI 1640 (phenol-red free) + 0.5% HSA, (b) 1 µM PMA, (c) 25µg/mL high oxLDL, (d) KC-PBS conditioned media, (e) KC-nLDL conditioned media, or (f) KC-Hox conditioned media. Each condition had 3 replicates. Plate was placed in the IncuCyte S3, and images were acquired every 10 minutes for 12 hours.

### Phagocytosis

Kupffer cells from 4 healthy donors were plated at 10^4^ cells/well in 96-weel plates in 10%FBS RPMI for 2h. Media was replaced by 0.5% human serum albumin RPMI plus: PBS, 50µg/mL nLDL or 50µg/mL HoxLDL (final concentrations), and 10µg of Incucyte^®^ pHrodo^®^ Red *E. coli* Bioparticles^®^ (Essen Biosciences) per well. Each condition had 4 replicates. For negative controls, some wells had only cells or bioparticles. Cells were analyzed in the IncuCyte S3, and images were acquired every 10 minutes for 5.5h.

### Image Quantification and Statistical Analyses

Chemotaxis quantification was performed by Incucyte^®^ Chemotaxis Analysis Software Module. Phagocytosis and NETosis events were quantified by IncuCyte^®^ basic analyzer software.

All statistical analyses and graphs were generated using GraphPad Prism version 8 for macOS (GraphPad Software www.graphpad.com). A Two-way ANOVA test was applied for Chemotaxis, Phagocytosis and NETosis. For the qPCR, we used Ordinary one-way ANOVA test.

### Luminex

Human Magnetic Luminex Assay (6-Plex) (R&D systems) was used to measure chemokines in the supernatant of M2-like macrophage cultures after 24h stimulation with PBS or HoxLDL. The supernatant was diluted at 1:2, and the experiment followed the manufacturer’s instructions. The chemokines analyzed were: CCL3/MIP-1 alpha (BR35), CCL4/MIP-1 beta (BR37), CXCL1/GRO alpha/KC/CINC-1 (BR77), CXCL2/GRO beta/MIP-2/CINC-3 (BR27), CXCL5/ENA-78 (BR52), and IL-8/CXCL8 (BR18).

## Results

### The Degree of Cholesterol Oxidation Drives Transcriptomic Changes in Human M2-Like Macrophages

We used the low-pass RNAseq approach to measure the changes of the highly expressed genes in human monocyte-derived macrophages exposed to native, medium oxidized- and highly oxidized LDL. We applied the *DESeq2* package (21) to identify differentially expressed genes (DEG), with significance threshold of FDR < 0.1 and fold change of at least 1.5 when compared to paired control (PBS) samples.

**Figure 1:**
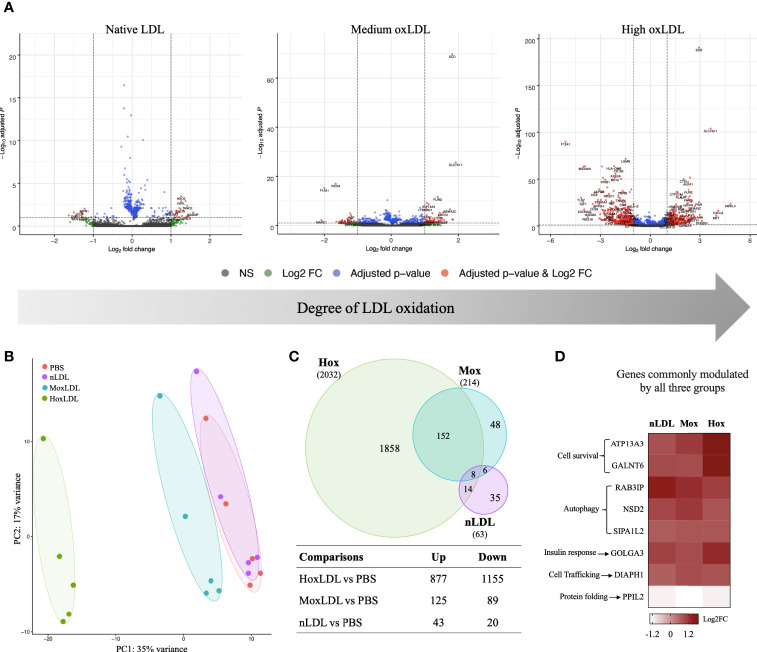
The degree of cholesterol oxidation drives transcriptomic changes in human M2-like macrophages. **(A)** The volcano plots display the changes in the M2-like macrophages gene expression after 24 hours of LDL-stimulation compared to cells stimulated by PBS *(n=5)*. Three types of LDL were used: non-oxidized (native), medium oxidized (MoxLDL), and highly oxidized (HoxLDL). The transcriptomic changes increase according to the degree of LDL oxidation. **(B)** The principal component analysis confirms the significance of transcriptomic changes induced by LDL oxidation. Ellipses indicate the confidence interval of groups. Axis percentages indicate variance contribution. **(C)** The Venn Diagram shows the overlapping genes in each group. Only 8 genes are commonly induced by LDL regardless of the oxidation degree, while 152 genes are stimulated due to LDL oxidation. The table shows the amount of upregulated and downregulated genes in the groups. **(D)** The common genes expressed due to LDL exposure are all upregulated and are related to cell survival, endoplasmic reticulum stability, response to insulin, and autophagy.

Our results showed that the LDL oxidation degree had different effects on the global gene expression of those cells. Cells incubated with highly oxidized LDL displayed the most dramatic changes (**Figures 1A, B**). Compared to unstimulated (PBS) cells (**Figure 1C**), native LDL (nLDL) induced 63 genes, with only 43 genes upregulated and 20 genes downregulated (top 40 genes on **Table S1**). Medium oxLDL (MoxLDL) stimulated 214 genes, with upregulation of 125 and downregulation of 89 genes (top 40 genes on **Table S2**). In the presence of highly oxidized LDL (HoxLDL), a total of 2032 genes were differentially expressed (top 40 genes on **Table S3**); surprisingly, more genes were downregulated (1155) than upregulated (877). All stimuli triggered a set of unique genes: 35 genes by nLDL, 48 genes by MoxLDL, and 1858 genes by hoxLDL. The 8 genes commonly modulated by nLDL, MoxLDL, and HoxLDL are related to cell survival, autophagy, response to insulin, cell trafficking and protein folding (**Figure 1D**).

### Pathway Analyses Identify HoxLDL-Induced Processes in Cholesterol Metabolism, Oxidative Stress Response, and Innate Inflammation in Macrophages

To identify a gene expression pattern that could distinguish cells stimulated by nLDL, MoxLDL, and HoxLDL, we evaluated the DEG lists (stimuli *vs* PBS) using the Ingenuity Pathway Analysis (IPA) biomarker tool. Then, the top 40 upregulated genes of each IPA biomarker list (i.e., nLDL *vs* PBS; MoxLDL *vs* PBS, and HoxLDL *vs* PBS) were used to construct gene networks using GeneMANIA (23). Genes that did not show connection by co-expression, co-localization, and genetic/physical interactions were filtered out. The remaining genes were then used to perform hierarchical clustering using Cluster v3 (25).

**Figure 2:**
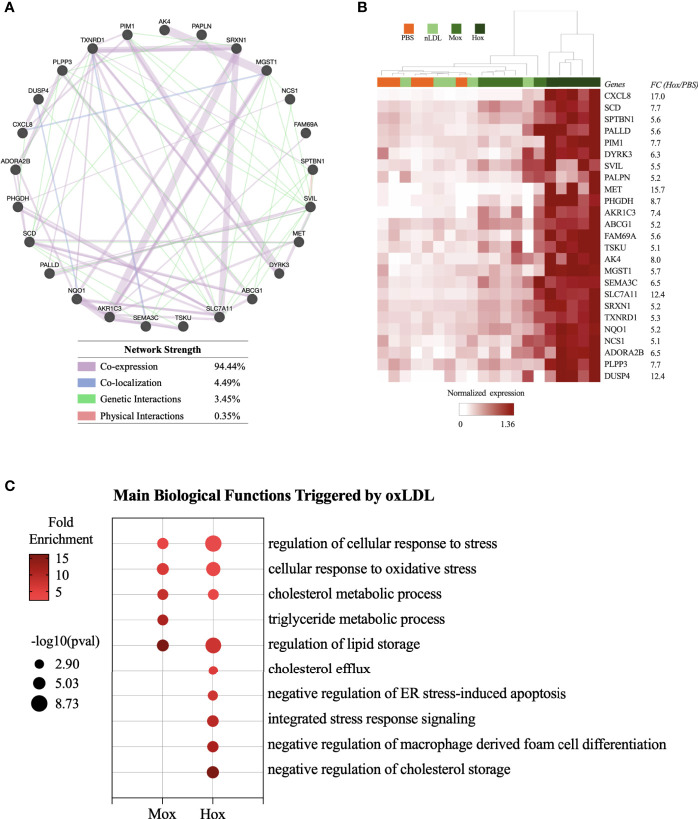
Transcriptome-based signature and biological processes triggered by HoxLDL stimulation. **(A)** Using the top 40 genes upregulated HoxLDL biomarker list, we performed a network test using GeneMANIA, which identified a strong relationship among the 25 genes. **(B)** In the heatmap, we can see that the hierarchical clustering of the 25 genes (HoxLDL group), group all the HoxLDL-stimulated samples together *(n=5)*. **(C)** Biological processes upregulated in M2-like cells exposed to MoxLDL and HoxLDL for 24 hours.

This network evaluation strategy identified a network of 25 genes in the MoxLDL (**Figure S1A**) data set and a network of 26 genes using the nLDL biomarkers list (**Figure S1B**). However, when clustered, these sets of genes did not group the samples according to the stimuli used (**Figures S1C, D**). In contrast, the network of 25 genes resulting from the HoxLDL dataset analysis showed not only robust network correlation (**Figure 2A**) but were also able to cluster the HoxLDL samples together (**Figure 2B**), confirming that the HoxLDL treatment induced a much stronger transcriptional change in the macrophages when compared to other stimuli.

The biological functions triggered by the LDL treatment were identified using the Gene Ontology platform. For these analyses, we used the lists of upregulated genes from each comparison (HoxLDL, MoxLDL, or nLDL *vs* PBS), and we excluded functions related to gene transcription and translation, and functions displaying less than 5 genes. For the cells treated with nLDL, no significant change was detected, while MoxLDL-treated cells showed an upregulation of lipid metabolism and oxidative stress. As expected, the main biological processes upregulated in HoxLDL-stimulated cells were also related to cholesterol metabolism and cellular stress, but surprisingly, these cells also exhibited significant inhibition of the foam cell formation and apoptotic processes (**Figure 2C** and **Figure S2**).

### HoxLDL Stimulation Shifts Peripheral and Hepatic Macrophages Toward an M4-Like Proinflammatory Phenotype

Moving forward with the DEG list obtained from HoxLDL-stimulated M2-like macrophages, we decided to investigate whether the detected transcriptional changes would affect the cells’ phenotype. Our analyses showed that the M2-like macrophages exposed to HoxLDL did not fully relate to the classical M1 and M2 phenotypes.

**Figure 3:**
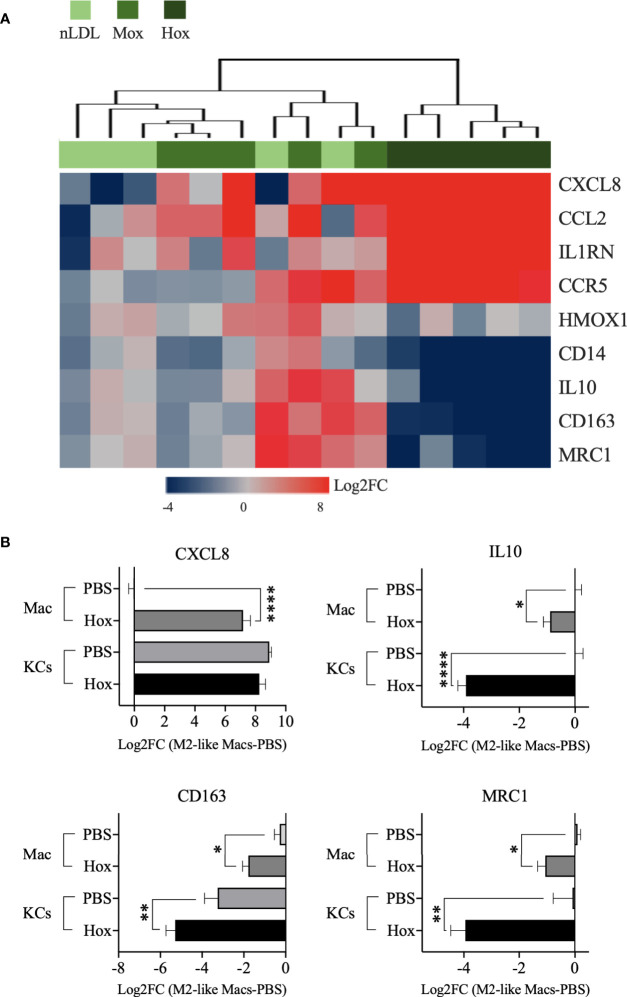
M2-like macrophages and Kupffer cells stimulated by HoxLDL switch to the M4-like proinflammatory phenotype. **(A)** Heatmap displays the M4-biomarkers expressed by M2-like macrophages exposed to nLDL, MoxLDL, and HoxLDL (values = Log2FC compared to PBS, *n=5*). Genes from the HoxLDL group follow the M4 profile, with upregulation of CXCL8, CCL2, CCR5, and IL1RN, and downregulation of CD14, CD163, HMOX1, MRC1, and IL10, suggesting that M2-like macrophages stimulated by HoxLDL change into the proinflammatory M4 phenotype. All the genes are significantly changed in the HoxLDL group. **(B)** Among the M4 biomarkers, we measured the expression of CXCL8, IL10, CD163, and MRC1 in human KCs with and without HoxLDL stimulation. Compared to *in vitro* M2-like differentiated macrophages *(n=5)*, KCs *(n=5)* are high constitutive producers of CXCL8, not changing significantly after HoxLDL exposure. However, the other common KCs’ markers were significantly downregulated after HoxLDL treatment, indicating that KCs may shift to the M4-like phenotype in the presence of HoxLDL as well. Data are presented as mean ± SEM. One way ANOVA test; *p <0.05, **p <0.01, ****p <0.0001.

We observed that HoxLDL induces a transcriptional shift in monocyte-derived macrophages consistent with the M4 macrophages (**Figure 3A**), with a mixture of M1 and M2 characteristics, as previously described in atherosclerotic plaques (27, 28). Based on the M4 macrophage characterization published by Gouwy et al. (6), which compared monocytes differentiated by CXCL4 and CXCL4L1 to resting M2-like macrophages, we evaluated the expression of M4 markers in M2-like macrophages treated with LDLs. For this purpose, we only considered the genes included in the DEG lists. Overall, HoxLDL-stimulated cells upregulated the expression of CXCL8, CCL2, CCR5, and IL1RN, and relative downregulation of CD14, CD163, HMOX1, MRC1, and IL10 when compared to resting M2-like macrophages. We also noticed the downregulation of several HLA genes, as expected for M4 macrophages, and surprisingly the decrease of TLRs’ gene expression as well (**Figure S3A**). In keeping with prior studies, these M4 macrophages derived from HoxLDL stimulation display expression patterns similar to CXCL4L1-exposed monocytes (**Figure S3B, C**). M2-like macrophages treated with MoxLDL or nLDL did not follow the M4-like gene expression pattern.

Furthermore, we evaluated some of these markers on human Kupffer cells (KCs) exposed to HoxLDL. We selected the scavenger receptor CD163 and mannose receptor MRC1 genes, which are commonly expressed by M1 and M2 KCs (29) but drastically downregulated in M4 cells, and the genes IL10 and CXCL8. HoxLDL-stimulated KCs significantly downregulated the transcription levels of IL10, CD163, and MRC1 (**Figure 3B**), while kept the CXCL8 expression unchanged but still high, similar to monocytes differentiated by CXCL4 (27).

### Kupffer Cells Phagocytic Capacity Is Reduced by HoxLDL Treatment

**Figure 4:**
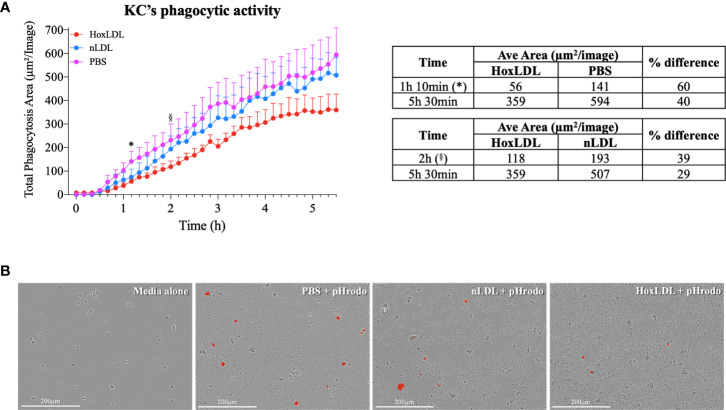
HoxLDL inhibits human Kupffer cells’ phagocytic activity. **(A)** HoxLDL exposure impairs KCs phagocytic activity starting at 1h 10min (*) statistically different from to unstimulated KCs-PBS, (§) statistically different from nLDL stimulated KCs *(n= 4)*. Two-way ANOVA test was used to detect statistical significance among time points. **(B)** Representative images from live-cell image assay, pHrodo red E-coli bioparticles^®^ become fluorescent inside the KCs’ lysosomes. Human KCs stimulated by HoxLDL image display less fluorescent-labeled cells, indicating phagocytic activity. Scale bar 200µm.

Phagocytosis is one of the crucial functions of macrophages and is significantly attenuated in M4 macrophages differentiated by CXCL4 (7). To analyze if HoxLDL-differentiated KCs’ phagocytic capacity was also impaired, we incubated KCs obtained from healthy livers with PBS, nLDL, or HoxLDL for 5.5 hours in the presence of heat-killed *E. coli* conjugated to a pH-sensitive fluorescent dye [pHrodo red E-coli bioparticles^®^] (**Figures 4A, B**). At 1h 10min, human KCs treated with HoxLDL significantly decreased their phagocytic capacity by 60% when compared to unstimulated KCs. When compared to nLDL treatment, HoxLDL significantly decreased phagocytosis by 39% at 2h. The inhibition of phagocytosis by HoxLDL was maintained for the entire time course. In contrast, phagocytic capacities of unstimulated KCs and nLDL-stimulated KCs were comparable.

### HoxLDL-Stimulated Kupffer Cells Demonstrate Increased Neutrophil Recruitment and NETosis Induction

**Figure 5:**
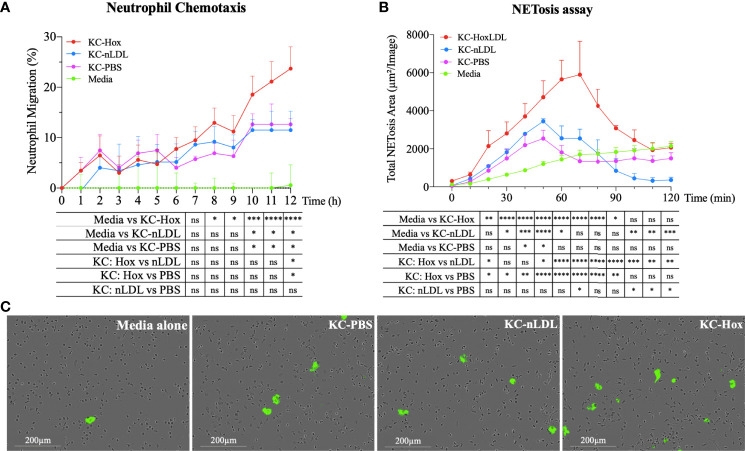
HoxLDL-derived M4 polarization enhances neutrophil recruitment and NETosis. **(A)** The live-cell imaging assay showed increased neutrophil recruitment after 8 hours in culture with HoxLDL-stimulated macrophages conditioned media (KC-Hox). Even though the conditioned media from nLDL-stimulated KCs (KC-nLDL) or unstimulated KCs (KC-PBS) alone also recruited neutrophils, HoxLDL has the strongest effect on neutrophils’ chemotaxis, achieving a migration peak at 12 hours *(n = 5)*. Two-way ANOVA test was used to detect statistical significance among time points, *p <0.05, **p <0.01, ***p <0.001, ****p <0.0001, ns, not significant. **(B)** KC-Hox conditioned media triggers NET formation, starting at 20 minutes and achieving a peak at 1h 10 minutes. KC-Hox’s NET induction was stronger than in conditioned media KC-PBS or KC-nLDL, lasting for almost 2 hours *(n=3)*. Two-way ANOVA test was used to detect statistical significance among time points, *p <0.05, **p <0.01, ***p <0.001, ****p <0.0001, ns, not significant. **(C)** representative images from NET induction assay. Cytotox green reagent^®^ was used to label expelled DNA: Unstimulated neutrophils (media alone), neutrophils incubated with KC-PBC, KC-nLDL, and KC-Hox at 1h 10min of the assay. Scale bar 200µm.

The secretion of cytokines and chemokines is another hallmark function of macrophages, which contributes to the recruitment and activation of other inflammatory cells. Considering that several chemokines produced by M4-like macrophages could recruit neutrophils (**Figures S4A, B**), we generated conditioned media from normal KCs incubated with PBS, nLDL, or HoxLDL for a live-cell image neutrophil chemotaxis assay. As shown in **Figure 5A**, conditioned media from HoxLDL-treated KCs (KC-Hox) recruited significantly more neutrophils than media from PBS- or nLDL-stimulated KCs (KC-PBS and KC-nLDL, respectively). The differences were significant at 8h, reaching a peak of migration at 12h. No significant difference was observed between KC-PBS and KC-nLDL recruitment.

In addition to neutrophil recruitment, we also analyzed the influence of the conditioned media on neutrophil extracellular trap formation (NETosis), a process that collectively includes the disintegration of nuclear and granule membranes and externalization of molecules including [e.g., MPO, double-stranded (ds)DNA, histones] (14–18) leading to inflammation-mediated injury. For that purpose, fresh isolated human neutrophils were incubated with serum-free conditioned media obtained from KCs incubated with PBS, nLDL or HoxLDL. NET formation was detected with Incucyte^®^ Cytotox Green Dye (Essen Biosciences), which yield fluorescence upon binding to the deoxyribonucleic acid (DNA) extruded by the neutrophils. Apoptotic neutrophils were excluded by IncuCyte^®^ basic analyzer software. KC-Hox triggered NET formation at 20 minutes, achieving a maximum effect at 1h 10min, and lasting for almost 2 hours (**Figures 5B, C**). KC-PBS and KC-nLDL had comparable results and showed much lower NET formation induction.

Even though it is known that cholesterol crystals may affect the behavior of neutrophils (17), HoxLDL alone did not have any effect on neutrophil recruitment or NETosis initiation (**Figures S5A, B**).

## Discussion

**Figure 6:**
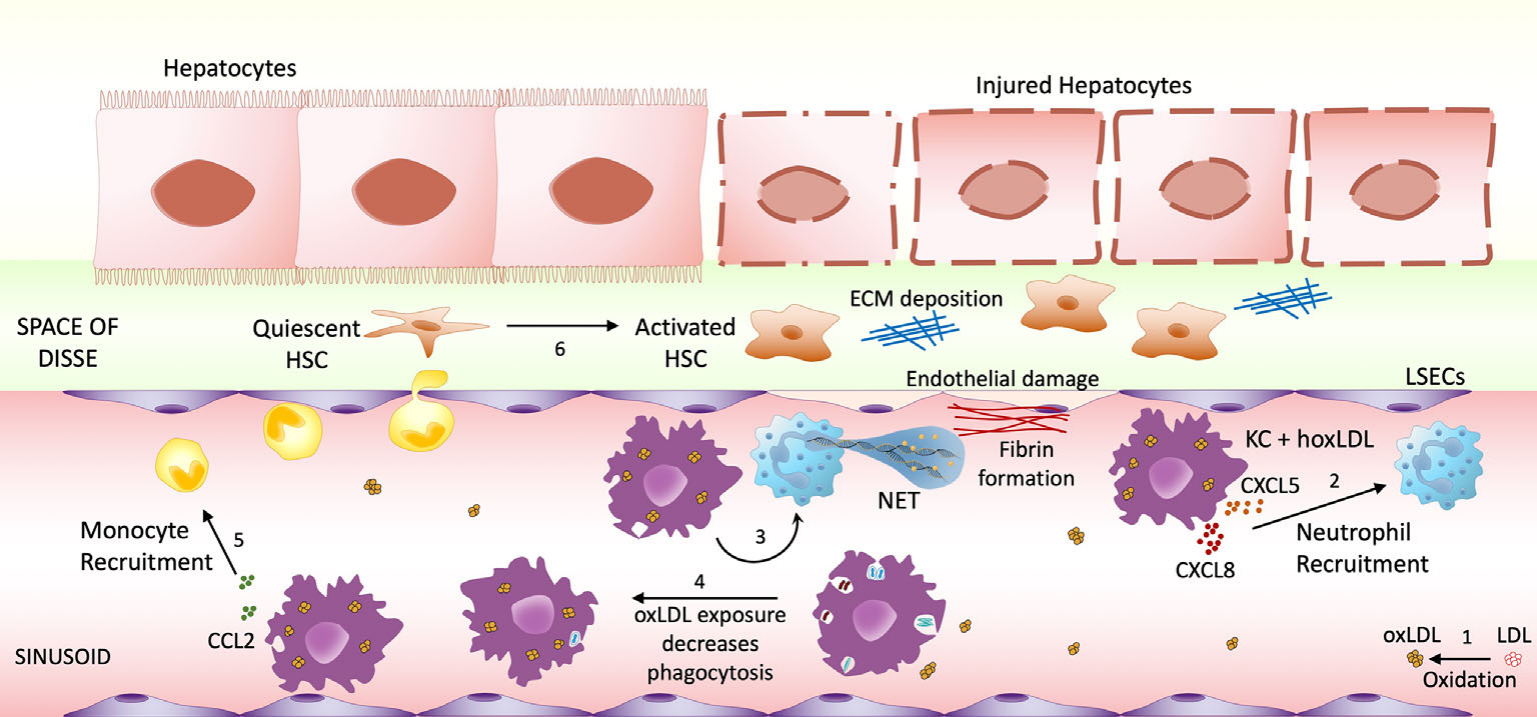
Highly oxidized LDL potential impact on NASH progression. (1) Low-density lipoprotein (LDL) can undergo oxidative modification. The increase of circulating oxLDL affects both infiltrating macrophages and sinusoid resident Kupffer cells. Once stimulated by LDL oxidized in high degrees, macrophages/KCs change their phenotype to M4-like proinflammatory phenotype, increasing the recruitment of neutrophils (2) and inducing NET formation (3), which will cause endothelial damage. M4 macrophages also decrease their phagocytic activity (4), possibly allowing the accumulation of endotoxin and microorganism. Another feature of these M4-like macrophages is the increased CCL2 production, possibly recruiting more monocytes to the inflammatory site (5). The combination of these events may contribute to Hepatic Stellate Cells (HSC) activation (6), resulting in hepatocyte injury and fibrosis progression.

This study provides novel insights into the multiple effects of oxLDL on macrophage responses and function, suggesting a new innate immune mechanism that may contribute to NASH progression (**Figure 6**). LDL is a notoriously unstable molecule, readily undergoing oxidation through non-enzymatic lipid peroxidation. The resulting neo-epitopes, termed “oxidation-specific-epitopes”, are endogenous “danger-associated molecular patterns” (DAMPs) that can be recognized by multiple innate pattern recognition receptors (30, 31). Prior work has identified the molecular mechanisms whereby oxLDL functions as an endogenous ligand that binds to CD36, triggering pathways necessary for inflammasome activation and IL-β secretion (11). Moreover, oxLDL’s slow clearance from lysosomes within macrophages may lead to cellular damage and inflammation (32, 33), and collective evidence points to unregulated uptake of oxLDL by Kupffer cells in NASH inflammation.

In this regard, we recently demonstrated in a murine model of NASH that the serum levels of oxLDL were proportional to the amount of dietary cholesterol included in the high-fat diet, but not when combined with a low-fat diet (2). Additionally, Kaikkonen et al. showed that elevated levels of serum oxLDL increase the risk of fatty liver development in humans and that LDL with high oxidation scores had the strongest association with NAFLD (12). Another recent study demonstrated that oxLDL accumulates in the portal vein of patients with NAFLD and co-localizes with activated macrophages (34, 35), strengthening the connection between oxLDL, macrophages, and liver inflammation.

The current study aimed to evaluate how LDL with different oxidation levels could affect peripheral macrophages’ and Kupffer cells’ functionality. Here, we showed a direct correlation between the degree of LDL oxidation and a global modification of M2-like macrophage transcriptome. Macrophages exposed to native LDL showed the smallest changes (only 63 genes), while medium oxLDL showed a moderate influence on the cell gene expression (214 genes). Highly oxLDL treatment had the highest impact, with a 32-fold increase in the number of genes differentially expressed when compared to the untreated cells. In addition, it was the only LDL that significantly triggered biological processes that prevent cholesterol storage, foam cell formation, and apoptosis. Our findings extend prior results demonstrating that stimulation of macrophages with oxidized (relative to native) LDL results in increased transcription of pro-inflammatory cytokines and chemokines in murine macrophages (36). In contrast to one report that derived macrophages using M-CSF and IL-10 (36), we found significant effects on cholesterol metabolism processes (i.e., cholesterol efflux, negative regulation of foam cell differentiation, and negative regulation of cholesterol storage). Our results suggest the possibility of adaptive responses within macrophages exposed to HoxLDL to attenuate the detrimental effects of foam cell formation (**Figure 2**). The discrepancy in results may be related to *in vitro* differences in cell culture, including macrophage derivation.

The traditional view that hepatic macrophages in NASH are predominantly pro-inflammatory (so-called M1) and contribute to disease by the production of cytokines such as IL-1α/β, TNFα, IL-6, and CCL2 (37) and recruitment of pro-inflammatory monocytes has been expanded to incorporate a role for tissue reparative macrophages (so-called M2) (38) associated with fibrosis development. Further underscoring the remarkable plasticity of macrophages, more recent data has identified M4-polarized macrophages, which are chemokine-induced, in other inflammatory diseases and atherosclerosis (6, 7, 27); however, no study has previously characterized M4 macrophages in the context of NAFLD. Our culture conditions generated M2-like macrophages (to mimic resting Kupffer cells) which were then stimulated with HoxLDL; the global transcriptional analyses identified key M4 biomarker genes CCR5, CXCL8, CCL2, IL1RN, CD14, CD163, HMOX1, MRC1, and IL10 that correlated with a public dataset defining M4 macrophages differentiated by CXCL4/CXCL4L1. Of interest, CXCL4 is known to be increased at the mRNA and serum levels in patients with NASH-related fibrosis and its genetic absence decreased infiltration of neutrophils in a murine model of liver injury (39), however, no CXCL4 expression was found in the stimulated macrophages in this study.

By treating normal human Kupffer cells with HoxLDL we were able to detect similar changes observed in the M2-like macrophages, with remarkable inhibition of IL10, MRC1, and CD163, but continuous high expression of CXCL8, suggesting these cells also acquire M4-like features when exposed to HoxLDL. Functionally, HoxLDL-differentiated KCs also resemble M4 macrophages, as their phagocytic capacity is suppressed when compared to resting KCs. Phagocytosis is an essential feature of KCs, which guarantees protection against the egress of endotoxin from the portal to the systemic circulation (40). Importantly, super-paramagnetic iron oxide (SPIO) magnetic resonance imaging studies have shown KC phagocytic dysfunction in NAFLD patients and NASH mice models (41, 42). KCs’ defective phagocytic function appears at the early stages of NAFLD, worsening with the progression from NAFLD to NASH, and it seems to be reversible after pioglitazone treatment, a drug that decreases LDL cholesterol levels (43, 44). Furthermore, another functional change observed in KCs treated with HoxLDL was the increase of neutrophil recruitment and boosting NET formation. It has been shown that the early stages of hepatic steatosis are characterized by intense recruitment of neutrophils that undergo NETosis, aggravating liver inflammation, and contributing to the progression of NASH, as well as hepatocellular carcinoma (45, 46).

In summary, our findings suggest that peripheral macrophages and Kupffer cells exposed to HoxLDL shift to M4-like phenotype and acquire new functions that may impact NAFLD progression. Taken together, these results contribute to better understand the innate immunity participation as well as the potential cholesterol contribution to NASH pathogenesis.

## Data Availability Statement

The datasets presented in this study can be found in online repositories. The names of the repository/repositories and accession number(s) can be found in the article/**Supplementary Material**.

## Ethics Statement

The studies involving human participants were reviewed and approved by University of Southern California. The patients/participants provided their written informed consent to participate in this study.

## Author Contributions

AM-M, LG-M, MK, and HR contributed to the conception and design of the study. AM-M executed the experiments and performed the statistical analysis. MS executed the bioinformatics and biostatistical analyses with the RNASeq data. AM-M, LG-M, and HR wrote the manuscript. All authors contributed to the article and approved the submitted version.

## Funding

This work was primarily supported by the National Institutes of Health grant numbers DK117004 and DK048522 (HR), and DK106491 (LG-M). MK was supported by the Intramural Research Program at NIAMS.

## Conflict of Interest

The authors declare that the research was conducted in the absence of any commercial or financial relationships that could be construed as a potential conflict of interest.
