# Supplementary material for: Cholesterol-Induced M4-Like Macrophages Recruit Neutrophils and Induce NETosis
Source: Front Immunol. 2021 May 3;12:671073. doi: 10.3389/fimmu.2021.671073 (PMC8126646; doi:10.3389/fimmu.2021.671073)
Supplement: Supplementary file 1 [file Table_1.docx]

Cholesterol-induced M4-like Macrophages recruit Neutrophils and induce NETosis.

Ana C. Maretti-Mira, Lucy Golden-Mason, Matthew P. Salomon, Mariana J. Kaplan, Hugo R. Rosen

**Contents List:**

Table S1

Table S2

Table S3

Figure S1

Figure S2

Figure S3

Figure S4

Figure S5

**Table S1.** Top 40 genes modulated by native LDL stimulation

| **Gene ID** | **symbol** | **log2FoldChange** | **pvalue** | **padj** |
| --- | --- | --- | --- | --- |
| ENSG00000127328 | RAB3IP | 1.57 | 4.9E-04 | 2.6E-02 |
| ENSG00000168874 | ATOH8 | 1.45 | 1.5E-03 | 7.0E-02 |
| ENSG00000132122 | SPATA6 | 1.44 | 1.6E-03 | 7.5E-02 |
| ENSG00000254681 | PKD1P5 | 1.43 | 1.2E-03 | 6.0E-02 |
| ENSG00000170921 | TANC2 | 1.42 | 1.7E-05 | 4.2E-03 |
| ENSG00000113494 | PRLR | 1.41 | 1.7E-03 | 7.6E-02 |
| ENSG00000172296 | SPTLC3 | 1.39 | 1.9E-03 | 8.7E-02 |
| ENSG00000136861 | CDK5RAP2 | 1.37 | 2.6E-06 | 1.2E-03 |
| ENSG00000189164 | ZNF527 | 1.31 | 2.5E-04 | 1.5E-02 |
| ENSG00000092871 | RFFL | 1.26 | 3.1E-06 | 1.3E-03 |
| ENSG00000123908 | AGO2 | 1.26 | 4.1E-07 | 3.1E-04 |
| ENSG00000140443 | IGF1R | 1.24 | 1.4E-04 | 1.0E-02 |
| ENSG00000162490 | DRAXIN | 1.23 | 4.4E-04 | 2.4E-02 |
| ENSG00000164574 | GALNT10 | 1.22 | 5.3E-05 | 7.9E-03 |
| ENSG00000137801 | THBS1 | 1.20 | 1.9E-03 | 8.4E-02 |
| ENSG00000165322 | ARHGAP12 | 1.16 | 5.5E-04 | 2.9E-02 |
| ENSG00000141219 | C17orf80 | 1.15 | 2.0E-03 | 9.0E-02 |
| ENSG00000101343 | CRNKL1 | 1.13 | 4.7E-04 | 2.5E-02 |
| ENSG00000174106 | LEMD3 | 1.13 | 2.7E-04 | 1.6E-02 |
| ENSG00000186260 | MRTFB | 1.10 | 1.1E-03 | 5.5E-02 |
| ENSG00000132305 | IMMT | -0.64 | 2.2E-03 | 9.8E-02 |
| ENSG00000171824 | EXOSC10 | -0.65 | 1.7E-03 | 7.7E-02 |
| ENSG00000132912 | DCTN4 | -0.71 | 7.1E-04 | 3.6E-02 |
| ENSG00000002549 | LAP3 | -0.73 | 1.8E-03 | 8.2E-02 |
| ENSG00000163171 | CDC42EP3 | -0.90 | 6.9E-04 | 3.5E-02 |
| ENSG00000175482 | POLD4 | -0.93 | 2.2E-03 | 9.6E-02 |
| ENSG00000106086 | PLEKHA8 | -0.95 | 1.6E-03 | 7.5E-02 |
| ENSG00000160194 | NDUFV3 | -1.06 | 2.0E-03 | 8.9E-02 |
| ENSG00000100023 | PPIL2 | -1.20 | 2.2E-03 | 9.8E-02 |
| ENSG00000171109 | MFN1 | -1.22 | 1.3E-04 | 9.7E-03 |
| ENSG00000183718 | TRIM52 | -1.27 | 2.1E-03 | 9.5E-02 |
| ENSG00000114737 | CISH | -1.28 | 2.1E-03 | 9.4E-02 |
| ENSG00000286070 | GGT1 | -1.29 | 8.6E-04 | 4.3E-02 |
| ENSG00000152457 | DCLRE1C | -1.30 | 4.7E-04 | 2.5E-02 |
| ENSG00000054219 | LY75 | -1.33 | 3.5E-04 | 2.0E-02 |
| ENSG00000161551 | ZNF577 | -1.44 | 8.1E-04 | 4.1E-02 |
| ENSG00000136895 | GARNL3 | -1.44 | 1.3E-03 | 6.0E-02 |
| ENSG00000178295 | GEN1 | -1.45 | 1.7E-03 | 7.7E-02 |
| ENSG00000083807 | SLC27A5 | -1.50 | 4.6E-04 | 2.5E-02 |
| ENSG00000168273 | SMIM4 | -1.63 | 1.2E-03 | 5.7E-02 |

**Table S2.** Top 40 genes modulated by MoxLDL stimulation

| **Gene ID** | **symbol** | **log2FoldChange** | **pvalue** | **padj** |
| --- | --- | --- | --- | --- |
| ENSG00000151012 | SLC7A11 | 1.92 | 1.8E-30 | 2.4E-26 |
| ENSG00000196139 | AKR1C3 | 1.84 | 3.4E-05 | 4.0E-03 |
| ENSG00000099194 | SCD | 1.82 | 8.6E-75 | 2.3E-70 |
| ENSG00000137261 | KIAA0319 | 1.82 | 1.9E-05 | 3.1E-03 |
| ENSG00000075223 | SEMA3C | 1.75 | 1.0E-10 | 2.4E-07 |
| ENSG00000147852 | VLDLR | 1.65 | 8.4E-04 | 3.2E-02 |
| ENSG00000197321 | SVIL | 1.57 | 8.7E-05 | 5.7E-03 |
| ENSG00000160179 | ABCG1 | 1.55 | 3.8E-09 | 5.0E-06 |
| ENSG00000029993 | HMGB3 | 1.50 | 1.3E-04 | 7.7E-03 |
| ENSG00000248323 | LUCAT1 | 1.47 | 2.3E-06 | 7.4E-04 |
| ENSG00000143217 | NECTIN4 | 1.46 | 2.6E-04 | 1.2E-02 |
| ENSG00000173706 | HEG1 | 1.44 | 4.8E-09 | 6.1E-06 |
| ENSG00000112902 | SEMA5A | 1.43 | 4.4E-04 | 1.9E-02 |
| ENSG00000152818 | UTRN | 1.41 | 2.4E-07 | 1.2E-04 |
| ENSG00000131437 | KIF3A | 1.41 | 1.7E-04 | 8.7E-03 |
| ENSG00000115657 | ABCB6 | 1.41 | 1.0E-03 | 3.9E-02 |
| ENSG00000147872 | PLIN2 | 1.38 | 4.9E-16 | 2.6E-12 |
| ENSG00000130396 | AFDN | 1.37 | 1.9E-03 | 6.5E-02 |
| ENSG00000182704 | TSKU | 1.35 | 9.1E-04 | 3.4E-02 |
| ENSG00000108641 | B9D1 | 1.33 | 1.4E-03 | 5.0E-02 |
| ENSG00000163606 | CD200R1 | -1.27 | 7.9E-04 | 3.1E-02 |
| ENSG00000167914 | GSDMA | -1.27 | 5.4E-04 | 2.2E-02 |
| ENSG00000104549 | SQLE | -1.28 | 3.0E-03 | 9.4E-02 |
| ENSG00000132514 | CLEC10A | -1.28 | 2.3E-03 | 7.4E-02 |
| ENSG00000198794 | SCAMP5 | -1.29 | 1.8E-03 | 6.1E-02 |
| ENSG00000128039 | SRD5A3 | -1.31 | 9.1E-06 | 1.9E-03 |
| ENSG00000178852 | EFCAB13 | -1.37 | 1.9E-03 | 6.5E-02 |
| ENSG00000100023 | PPIL2 | -1.37 | 5.3E-04 | 2.2E-02 |
| ENSG00000152457 | DCLRE1C | -1.37 | 2.4E-04 | 1.2E-02 |
| ENSG00000175857 | GAPT | -1.39 | 3.0E-03 | 9.4E-02 |
| ENSG00000254703 | SENCR | -1.44 | 2.3E-03 | 7.4E-02 |
| ENSG00000072858 | SIDT1 | -1.46 | 1.5E-04 | 8.5E-03 |
| ENSG00000120738 | EGR1 | -1.47 | 1.3E-04 | 7.7E-03 |
| ENSG00000128394 | APOBEC3F | -1.48 | 1.3E-03 | 4.7E-02 |
| ENSG00000104921 | FCER2 | -1.48 | 1.2E-03 | 4.3E-02 |
| ENSG00000254860 | TMEM9B-AS1 | -1.53 | 4.2E-04 | 1.8E-02 |
| ENSG00000171241 | SHCBP1 | -1.64 | 6.3E-04 | 2.6E-02 |
| ENSG00000155659 | VSIG4 | -1.66 | 1.1E-21 | 9.3E-18 |
| ENSG00000124491 | F13A1 | -2.00 | 1.0E-19 | 6.8E-16 |
| ENSG00000186205 | MARC1 | -2.08 | 1.0E-04 | 6.4E-03 |

**Table S3.** Top 40 genes modulated by HoxLDL stimulation

| **Gene ID** | **symbol** | **log2FoldChange** | **pvalue** | **padj** |
| --- | --- | --- | --- | --- |
| ENSG00000172548 | NIPAL4 | 4.83 | 1.1E-26 | 4.1E-24 |
| ENSG00000169429 | CXCL8 | 4.09 | 6.2E-19 | 1.2E-16 |
| ENSG00000105976 | MET | 3.97 | 1.8E-13 | 2.1E-11 |
| ENSG00000151012 | SLC7A11 | 3.63 | 1.7E-108 | 2.0E-104 |
| ENSG00000092621 | PHGDH | 3.12 | 1.7E-07 | 7.5E-06 |
| ENSG00000137261 | KIAA0319 | 3.09 | 1.5E-11 | 1.4E-09 |
| ENSG00000162433 | AK4 | 3.00 | 3.6E-14 | 4.7E-12 |
| ENSG00000099194 | SCD | 2.94 | 1.4E-195 | 3.4E-191 |
| ENSG00000137193 | PIM1 | 2.94 | 1.8E-28 | 7.3E-26 |
| ENSG00000196139 | AKR1C3 | 2.88 | 3.9E-08 | 2.0E-06 |
| ENSG00000075223 | SEMA3C | 2.70 | 3.7E-24 | 1.2E-21 |
| ENSG00000143479 | DYRK3 | 2.67 | 6.3E-12 | 6.2E-10 |
| ENSG00000135253 | KCP | 2.62 | 3.1E-13 | 3.5E-11 |
| ENSG00000107130 | NCS1 | 2.60 | 8.2E-15 | 1.2E-12 |
| ENSG00000162407 | PLPP3 | 2.53 | 8.8E-30 | 4.1E-27 |
| ENSG00000008394 | MGST1 | 2.52 | 1.5E-10 | 1.1E-08 |
| ENSG00000115306 | SPTBN1 | 2.49 | 1.4E-28 | 5.9E-26 |
| ENSG00000129116 | PALLD | 2.48 | 9.8E-13 | 1.1E-10 |
| ENSG00000154511 | DIPK1A | 2.47 | 8.7E-09 | 4.9E-07 |
| ENSG00000106853 | PTGR1 | 2.46 | 9.3E-21 | 2.3E-18 |
| ENSG00000174600 | CMKLR1 | -2.94 | 2.0E-39 | 1.5E-36 |
| ENSG00000113532 | ST8SIA4 | -2.98 | 2.9E-31 | 1.5E-28 |
| ENSG00000133561 | GIMAP6 | -3.00 | 4.1E-10 | 3.0E-08 |
| ENSG00000170271 | FAXDC2 | -3.02 | 2.0E-15 | 3.0E-13 |
| ENSG00000105609 | LILRB5 | -3.08 | 6.5E-43 | 7.4E-40 |
| ENSG00000132514 | CLEC10A | -3.08 | 1.2E-10 | 9.3E-09 |
| ENSG00000171659 | GPR34 | -3.10 | 5.5E-27 | 2.1E-24 |
| ENSG00000179583 | CIITA | -3.15 | 3.0E-31 | 1.5E-28 |
| ENSG00000125810 | CD93 | -3.28 | 3.0E-39 | 2.3E-36 |
| ENSG00000019991 | HGF | -3.36 | 3.4E-39 | 2.5E-36 |
| ENSG00000178562 | CD28 | -3.38 | 1.4E-23 | 4.4E-21 |
| ENSG00000182162 | P2RY8 | -3.47 | 1.1E-26 | 4.0E-24 |
| ENSG00000167914 | GSDMA | -3.60 | 3.4E-17 | 5.9E-15 |
| ENSG00000150681 | RGS18 | -3.77 | 3.4E-12 | 3.5E-10 |
| ENSG00000110077 | MS4A6A | -3.94 | 5.7E-68 | 2.3E-64 |
| ENSG00000282608 | ADORA3 | -3.98 | 9.3E-21 | 2.3E-18 |
| ENSG00000138449 | SLC40A1 | -4.03 | 2.0E-67 | 6.2E-64 |
| ENSG00000017427 | IGF1 | -4.04 | 1.8E-29 | 8.2E-27 |
| ENSG00000196664 | TLR7 | -4.17 | 1.4E-33 | 8.2E-31 |
| ENSG00000124491 | F13A1 | -5.10 | 2.0E-94 | 1.6E-90 |


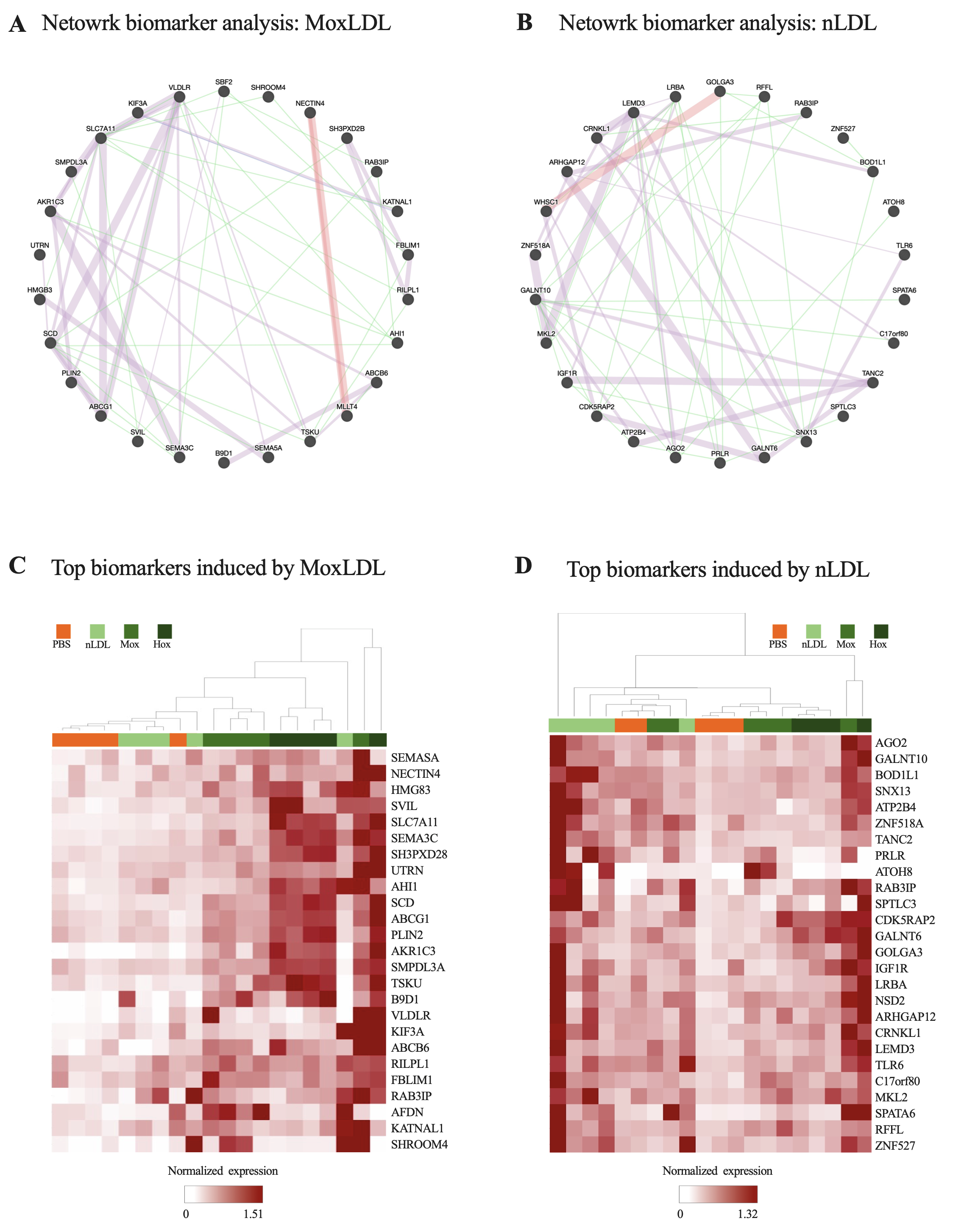


**Figure S1.** Search for biomarkers induced by MoxLDL and nLDL. Using the top 40 genes upregulated in the respective IPA biomarker lists, GeneMANIA elected 25 genes for MoxLDL-stimulated samples (A), and 26 genes for nLDL-treated cells (B). Even though all the genes showed a strong network connection, they were not able to group the samples according to the stimuli (C-D).


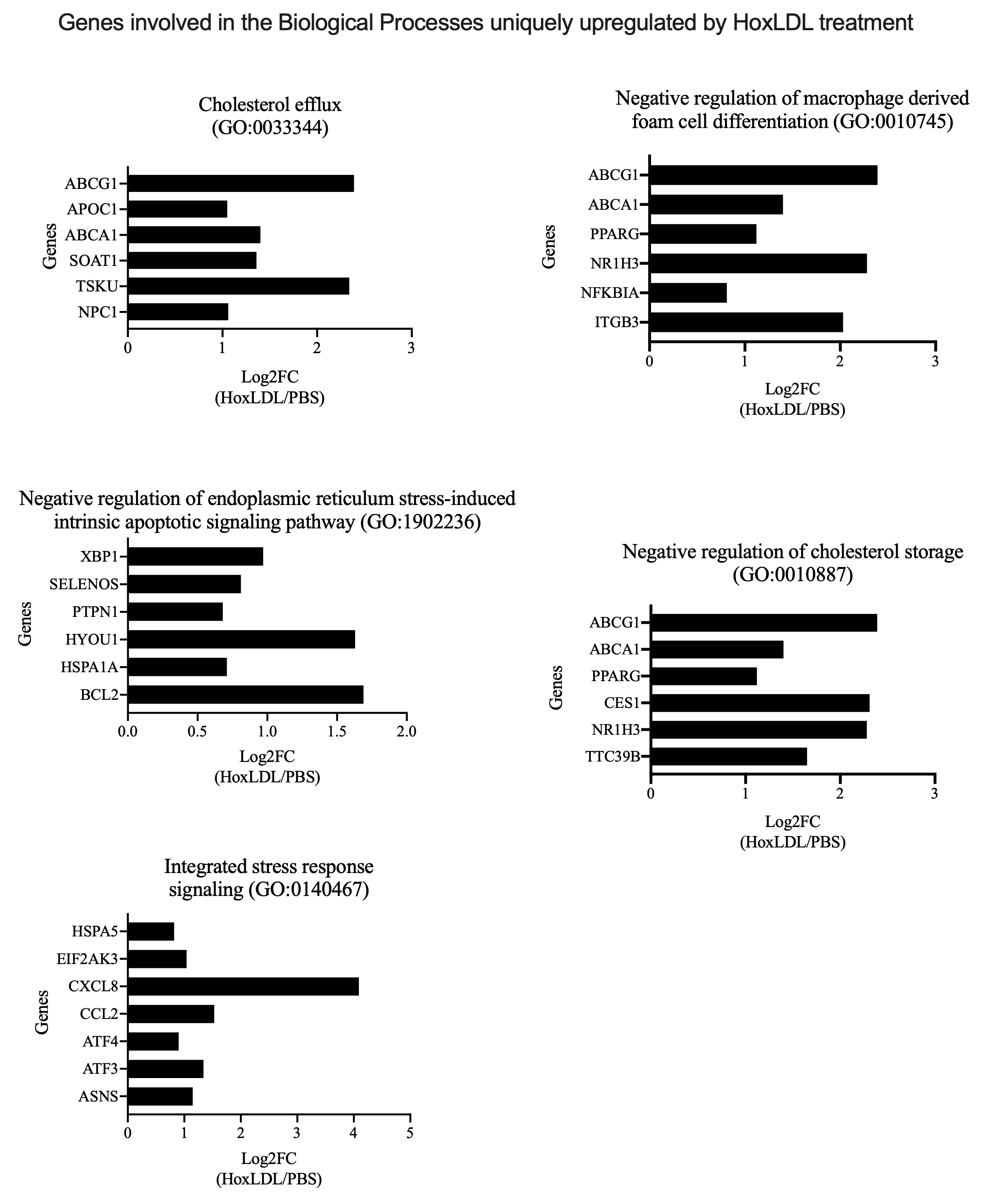


**Figure S2.** Gene sets of the main biological processed uniquely upregulated by HoxLDL. Using Gene Ontology platform, five significant biological processes were upregulated in M2-like macrophages stimulated by HoxLDL when compared to controls (PBS). The graphs show the modulation of the genes involved in these processes.


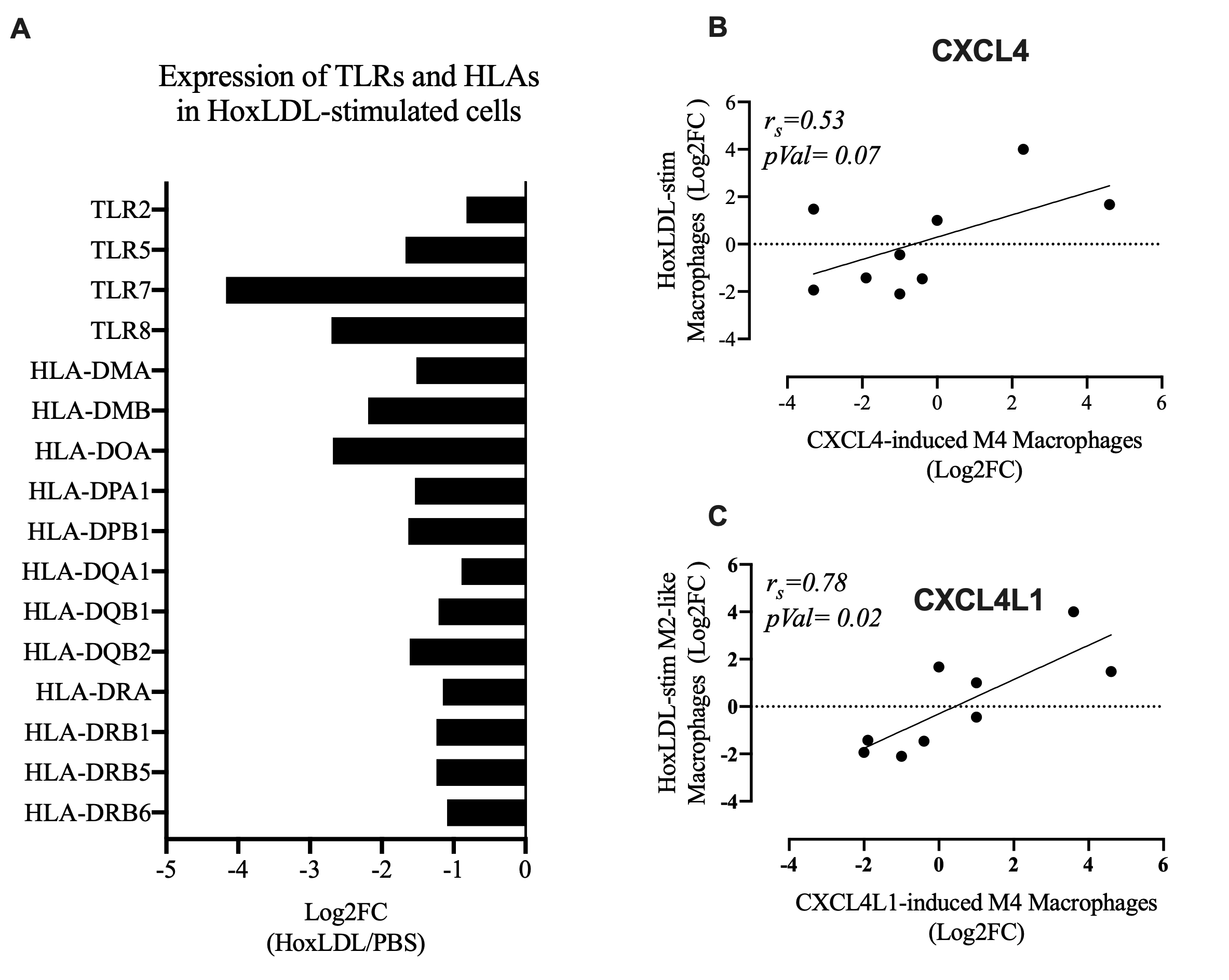


**Figure S3.** HoxLDL effects on M2-like macrophages are comparable to the M4 polarization induced by CXCL4 and CXCL4L1. **A**. Several TLR and HLA genes are downregulated in HoxLDL-stimulated M2-like macrophages, corroborating with the observation by other authors that M4 macrophages display low HLA-DR expression. **B** and **C** show the Spearman correlation plots comparing the expression of the keys M4 biomarker genes CCR5, CXCL8, CCL2, IL1RN, CD14, CD163, HMOX1, MRC1, and IL10. The comparisons were done using HoxLDL-stimulated cells and the published dataset from M4 macrophages differentiated by CXCL4 (B) or CXCL4L1 (C).

**
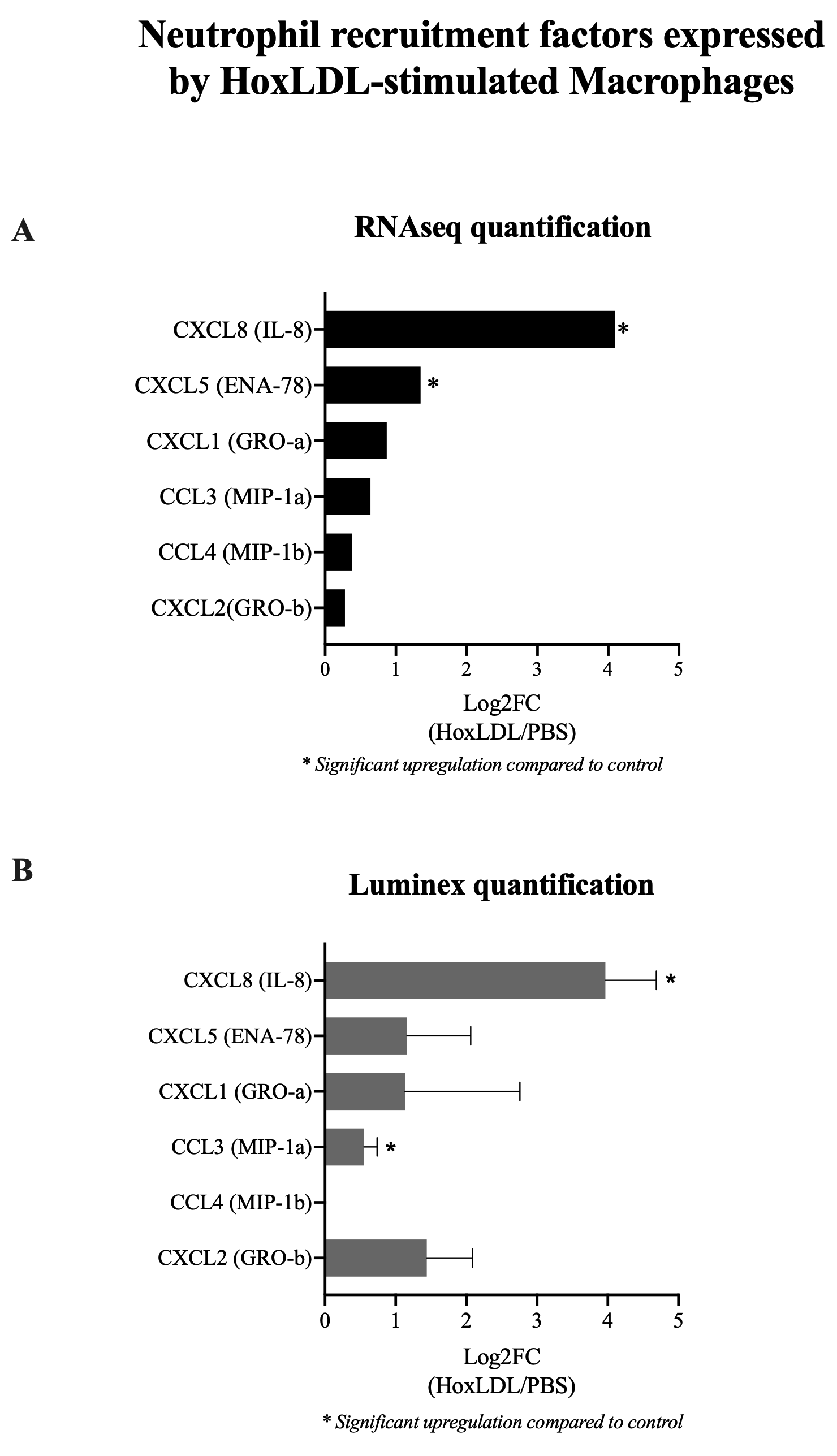
**

**Figure S4.** M4-like macrophages resulting from HoxLDL stimulation express several neutrophil chemoattractants. **(A)** According to RNAseq results, the chemokines with significant upregulation are CXCL8 (IL8) and CXCL5 (ENA-78). Data are presented in mean. Significance was determined by *DESeq2*. **(B)** Protein expression was evaluated at supernatants using Luminex technology. CXCL8 is the chemokine detected with the highest level (n= 5). Data are presented in mean (+) SEM, and significance tested using Paired T test (two-tailed), * p < 0.05.


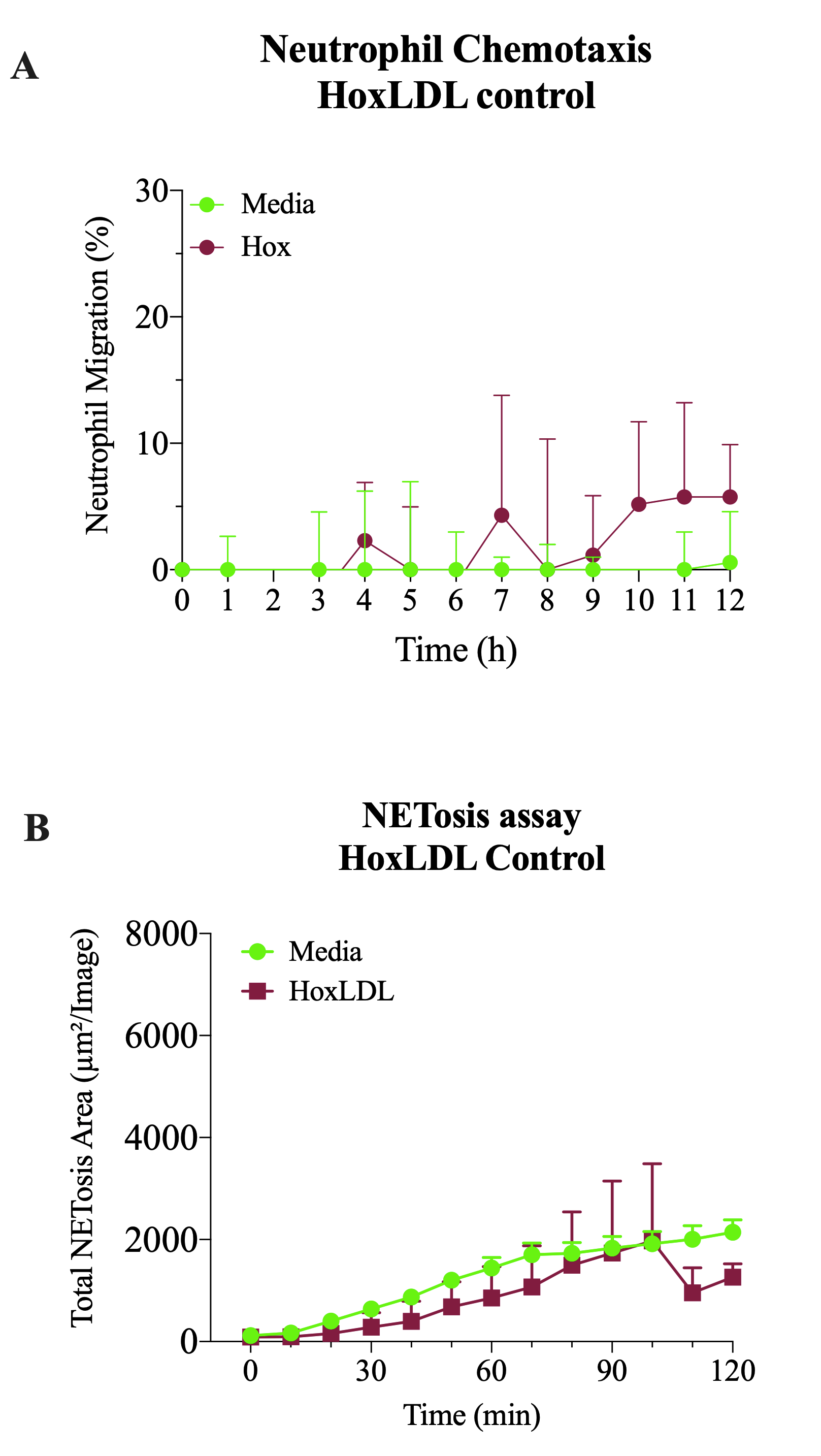


**Figure S5.** Effects of HoxLDL alone on neutrophil migration **(A)** and NETosis **(B)**. Results are comparable to media alone.
